# Increased Serum Levels of Brain-Derived Neurotrophic Factor Contribute to Inflammatory Responses in Patients with Rheumatoid Arthritis

**DOI:** 10.3390/ijms22041841

**Published:** 2021-02-12

**Authors:** Ning-Sheng Lai, Hui-Chun Yu, Hsien-Yu Huang Tseng, Chia-Wen Hsu, Hsien-Bin Huang, Ming-Chi Lu

**Affiliations:** 1 Division of Allergy, Immunology and Rheumatology, Dalin Tzu Chi Hospital, Buddhist Tzu Chi Medical Foundation, Dalin, Chiayi 62247, Taiwan; Q12015@tzuchi.com.tw (N.-S.L.); junvsusagi@gmail.com (H.-C.Y.); dm248871@tzuchi.com.tw (H.-Y.H.T.); dl50299@tzuchi.com.tw (C.-W.H.); 2 School of Medicine, Tzu Chi University, Hualien City 97071, Taiwan; 3 Department of Life Science and Institute of Molecular Biology, National Chung Cheng University, Minxiong, Chiayi 62130, Taiwan; biohbh@ccu.edu.tw

**Keywords:** BDNF, rheumatoid arthritis, JNK, T cells, anxiety, proinflammatory cytokines

## Abstract

The aim of this study is to investigate the role of brain-derived neurotrophic factor (BDNF) in the inflammatory responses in patients with rheumatoid arthritis (RA). Serum levels of BDNF and the precursor form of BDNF (proBDNF) from 625 RA patients and 40 controls were analyzed using enzyme-linked immunosorbent assay. Effects of BDNF on the mitogen-activated protein kinase pathway were analyzed by Western blotting. Microarray analysis was conducted to search BDNF regulated gene expression in Jurkat cells, and the differentially expressed genes were validated using T cells from patients with RA and controls. Serum BDNF levels were significantly elevated in patients with RA compared with the controls. Low serum BDNF levels were found in RA patients with anxiety or receiving biologics treatment. BDNF (20 ng/mL) enhanced the phosphorylation of ERK, JNK, and c-Jun, but suppressed the phosphorylation of p38, whereas BDNF (200 ng/mL) enhanced the phosphorylation of ERK and p38. After validation, the expression of *CAMK2A*, *MASP2*, *GNG13*, and *MUC5AC*, regulated by BDNF and one of its receptors, *NGFR*, was increased in RA T cells. BDNF increased the *IL-2*, *IL-17*, and *IFN-γ* expression in Jurkat cells and IL-2 and IFN-γ secretion in activated peripheral blood mononuclear cells.

## 1. Introduction

Rheumatoid arthritis (RA) is a chronic systemic disease characterized by persisting joint inflammation. In addition to articular manifestations, patients with RA have an increased incidence of developing depression, which is itself a risk factor for developing RA [1]. Decreased brain-derived neurotrophic factor (BDNF) expression is well-known to play a critical role in the pathogenesis of depression [2]. BDNF is initially synthesized as the precursor form of BDNF (proBDNF) in neurons and glia, which is cleaved to mature BDNF intracellularly or extracellularly to release mature BDNF [3].

Previous research suggested that the increased serum levels of proBDNF or decreased BDNF/proBDNF ratio could be a serum marker for depression [4,5]. However, few studies have investigated the serum levels of BDNF in patients with RA, and the results were conflicting. Low serum BDNF levels were observed in RA patients with depression compared to RA patients without depression [6]. Grimsholm et al. found that serum BDNF was elevated in 18 patients with RA compared with controls, and their BDNF levels were declined after receiving anti-TNF treatment [7]. Whether patients with RA have elevated serum BDNF levels remains to be elucidated. Furthermore, there were no data available for serum proBDNF levels and BDNF/proBDNF ratio in patients with RA.

Recent evidence suggested that there are close interactions and communications between the immune system and the nervous system. For example, melatonin, which is secreted by the pineal gland, could suppress inflammation in preclinical models of RA [8,9]. We speculated that BDNF and it’s signaling pathway could play a role in the inflammatory response of RA. Therefore, the aim of this study was to investigate the serum levels of BDNF, proBDNF and their ratio in RA patients compared with controls and to search for significant clinical manifestations associated with BDNF. We also searched for the expression of genes that are regulated by BDNF and clarified the potential roles of BDNF in the inflammatory response using T cells and its respective cell line, Jurkat cells.

## 2. Results

### 2.1. Serum BDNF, proBDNF Levels, and proBDNF/BDNF Ratio in Patients with RA and Controls

**Figure 1:**
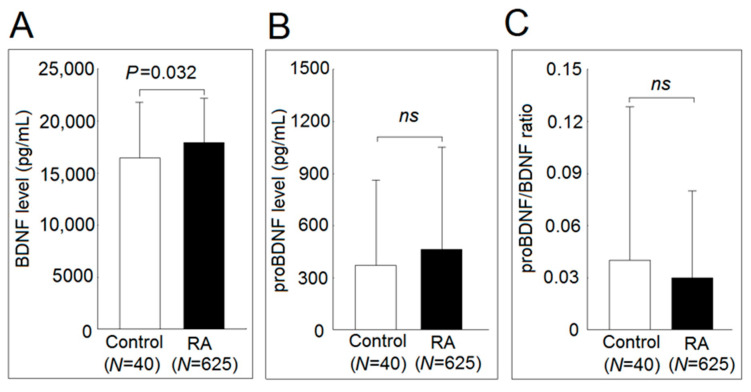
Serum brain-derived neurotrophic factor (BDNF), the precursor form of BDNF (proBDNF) concentration, and proBDNF/BDNF ratio in healthy controls and patients with rheumatoid arthritis. Serum levels of (**A**) BDNF, and (**B**) proBDNF concentration and (**C**) proBDNF/BDNF ratio in 40 healthy controls and 625 patients with rheumatoid arthritis. Serum levels of BDNF and proBDNF were measured by enzyme-linked immunosorbent assay (ELISA). After adjusting for age and sex in the multiple linear regression analysis, the BDNF levels remained significantly (*p* = 0.033) elevated in serum from patients with rheumatoid arthritis compared with those from controls. (*ns* not significant; * *p* < 0.05).

A total of 625 patients (78.2% female) with RA aged 62.0 ± 13.5 years and 40 controls (67.5% female) aged 62.2 ± 14.1 years were enrolled in this study. The demographic, clinical, and psychological variables of study patients with RA are presented in our earlier study [10]. Ninety-six subjects (15.4%) were classified as having depression, and 65 subjects (10.4%) were classified as having anxiety. Patients with RA had significantly elevated serum BDNF levels compared with those of the controls (17.9 ± 4.2 ng/mL vs. 16.4 ± 5.3 ng/mL, *p* = 0.032). There were no statistically significant differences between the serum proBDNF levels (464 ± 583 pg/mL vs. 372 ± 487 pg/mL; *p* = 0.328) and proBDNF/BDNF ratio (0.03 ± 0.05 vs. 0.04 ± 0.09; *p* = 0.370) between patients with RA and controls (Figure 1). After adjusting for age and sex, the serum BDNF levels remained significantly (*p* = 0.033) elevated in patients with RA compared with the controls.

### 2.2. Correlation of Clinical Parameters with Serum BDNF Levels in Patients with RA

The association of various clinical parameters and serum levels of BDNF in patients with RA was analyzed in Table 1. In the univariate analysis, serum BDNF levels were inversely associated with an increased swollen joint count over 28 joints (*p* = 0.023) and age (*p* = 0.015). RA patients with anxiety (*p* = 0.003), receiving biologics (including etanercept, adalimumab, golimumab, abatacept, tocilizumab, tofacitinib, and rituximab) (*p* = 0.01), retired (*p* = 0.006), and had a disease duration of more than 5 years showed significantly lower serum level of BDNF. In the multiple regression analysis, only RA patients with anxiety (*p* = 0.002) and receiving biologics (*p* = 0.020) were significantly associated with lower serum levels of BDNF.

### 2.3. Expression of BDNF Receptors and Effects of BDNF in Mitogen-Activated Protein Kinase Phosphorylation and Cell Proliferation

**Figure 2:**
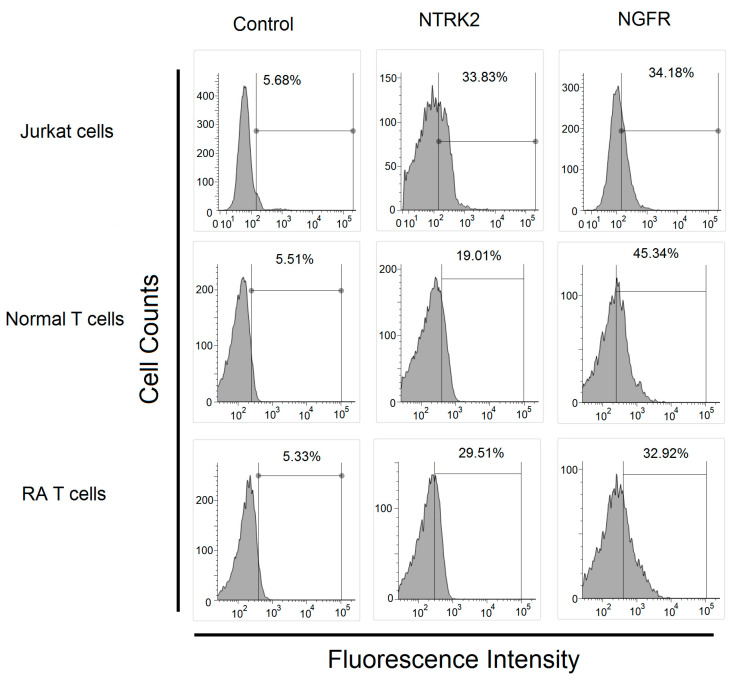
Expression of BDNF receptor in Jurkat cells and T cells from healthy controls and patients with rheumatoid arthritis. The expression of BDNF receptors, including neurotrophic receptor tyrosine kinase 2 (NTRK2) and nerve growth factor receptor (NGFR) in Jurkat cells, T cells from healthy controls and patients with rheumatoid arthritis analyzed using flow cytometry.

We confirmed that two BDNF receptors-NTRK2 and NGFR were expressed on T cells from patients with RA and controls and Jurkat cells (Figure 2).

**Figure 3:**
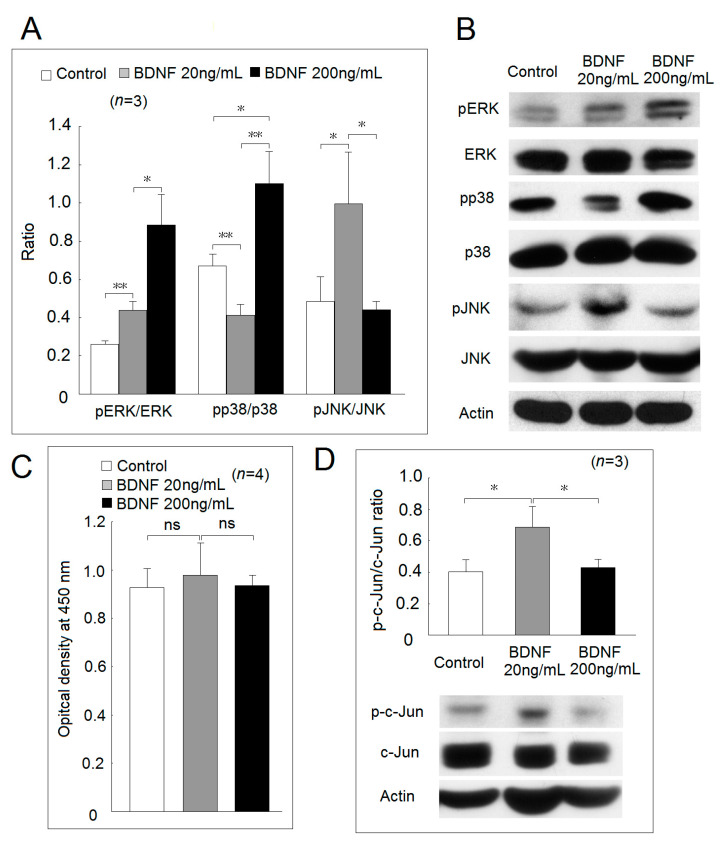
Effect of different concentrations of BDNF in mitogen-activated protein kinase (MAPK) phosphorylation and cell proliferation. (**A**) The phosphorylation ratio of p38, extracellular signal-regulated kinases (ERK), and c-Jun N-terminal kinases (JNK) in Jurkat cells after cocultured in culture medium with a low concentration BDNF (20 ng/mL) or a high concentration BDNF (200 ng/mL) for 48 h. (**B**) a representative case. (**C**) The viability and proliferation of Jurkat cells after cocultured in culture medium with a low and a high concentration of BDNF for 48 h analyzed by WST-1 cell proliferation assay. (**D**) The phosphorylation ratio of c-Jun in Jurkat cells after cocultured in culture medium with a low and a high concentration BDNF for 48 h (*ns*, not significant; * *p* < 0.05; ** *p* < 0.01).

We further investigated the downstream signaling pathway of BDNF. The phosphorylated ratio of ERK, p38, and JNK in Jurkat cells after culturing with a high concentration of BDNF (200 ng/mL), low concentration of BDNF (20 ng/mL) for 48 h or culture medium only as the control group was measured (Figure 3A,B). We found that BDNF could increase the phosphorylation of ERK in a dose-dependent manner. The phosphorylated ratio of p38 was decreased significantly in Jurkat cells cocultured with a low concentration of BDNF compared with the controls (*p* = 0.006). The phosphorylated ratio of p38 was elevated in Jurkat cells cocultured with a high concentration of BDNF compared with the controls (*p* = 0.013) or low concentration BDNF group (*p* = 0.002). Finally, the phosphorylation ratio of JNK was found to be significantly elevated in the Jurkat cells cocultured with a low concentration BDNF compared with the controls (*p* = 0.041) or a high concentration BDNF group (*p* = 0.025). There were no differences in the phosphorylation ratio of JNK between the high concentration BDNF group and the controls. For the possible effect of ERK activation, we found that the addition of a low or high concentration BDNF did not affect the Jurkat cell viability and proliferation (Figure 3C). We also confirmed that the activation of JNK led to the increased phosphorylation of c-Jun, a downstream molecule of the JNK signaling pathway in the low concentration BDNF group (Figure 3D).

### 2.4. Investigation of the BDNF-Regulated Gene Focusing on Inflammation-Related Proteins and Their Expression in Patients with RA

**Figure 4:**
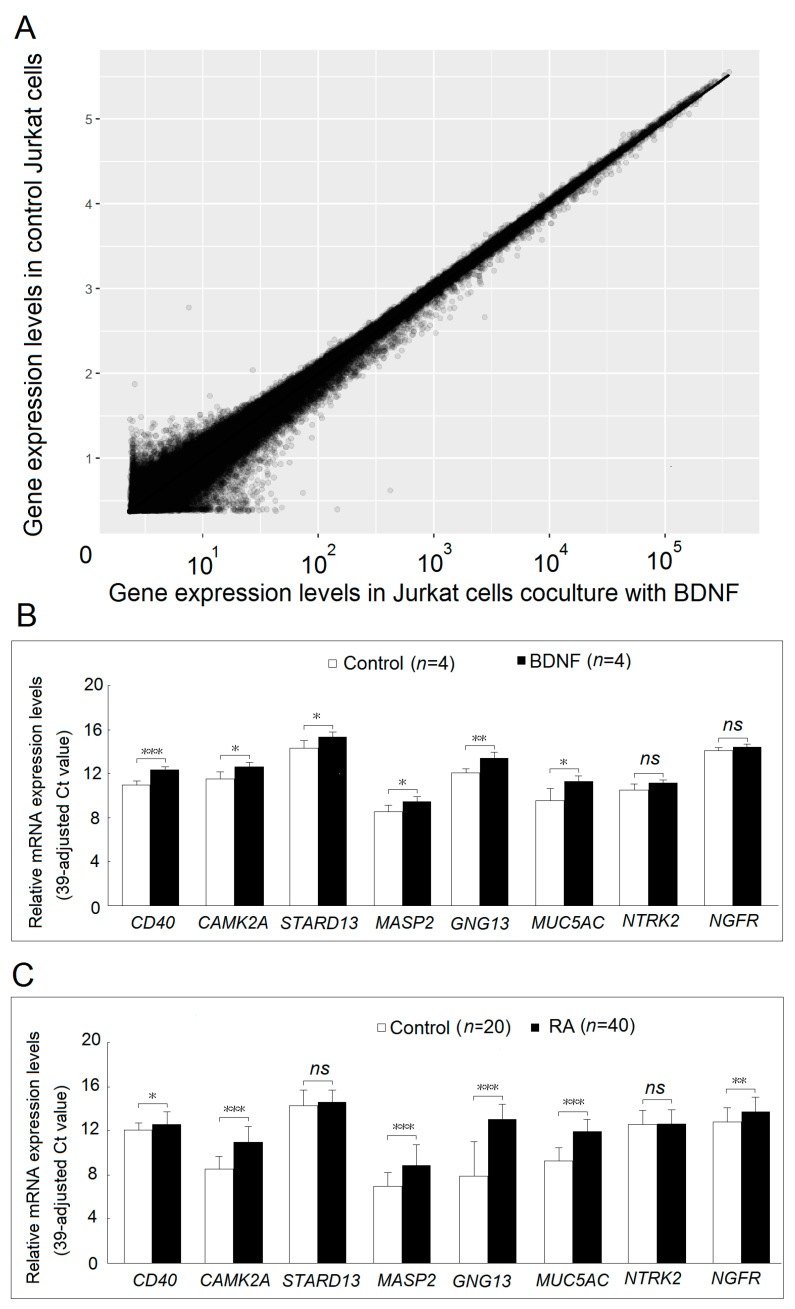
Identification and validation of BDNF regulated gene and its expression levels in T cells from patients with rheumatoid arthritis and healthy controls. (**A**) Expression profiles of mRNAs in Jurkat cells cocultured with BDNF 200 ng/mL or culture medium for 48 h were evaluated using microarray analysis. Each scatter spot represents the mean raw signal of mRNA in three repeats of each treatment. (**B**) Six genes, including *CD40*, *CAMK2A*, *STARD13*, *MASP2*, *GNG13*, and *MUC5AC*, which are related to the inflammatory pathway, were validated using real-time PCR. The expression levels of the BDNF receptors: *NTRK2* and *NGFR* were compared in Jurkat cells cocultured with BDNF 200 ng/mL or culture medium for 48 hours. (**C**) The expressed levels of *CD40*, *CAMK2A*, *STARD13*, *MASP2*, *GNG13*, *MUC5AC*, *NTRK2*, and *NGFR* were compared in T cells from healthy controls and patients with rheumatoid arthritis. (*ns*, not significant; * *p* < 0.05; ** *p* < 0.01; *** *p* < 0.001).

After microarray analysis, we found that the expression of 77 protein-coding genes was significantly decreased, and 142 protein-coding genes were significantly increased in Jurkat cells after cocultured with BDNF 200 ng/mL for 48 h compared with the controls (Figure 4A). We selected genes that could potentially involve in inflammatory responses and validated them using real-time PCR. We found that the expression of *Cluster of differentiation 40 (CD40)*, *Calcium/Calmodulin-Dependent Protein Kinase II Alpha (CAMK2A), StAR Related Lipid Transfer Domain-Containing 13 (STARD13)*, *Mannan-binding lectin serine protease 2 (MASP2), G Protein Subunit Gamma 13 (GNG13)* and *Mucin 5AC, Oligomeric Mucus/Gel-Forming (MUC5AC)* were significantly higher after validation (Figure 4B). The gene expression of *NTRK2* and *NGFR* did not differ in the microarray analysis; we checked their expression levels in the validation step by real-time PCR. As expected, their expression was not different in Jurkat cells after cocultured with BDNF 200 ng/mL for 48 h compared with the controls. We then obtained T cells from an additional 40 patients with RA and 20 healthy controls. The demographic and clinical data of the patients with RA and controls are shown in Table 2. There were no differences in age or sex between the two groups. Among the BDNF-regulated genes, we found that the expression of *CD40, CAMK2A, MASP2, GNG13*, and *MUC5AC* was significantly increased in T cells from patients with RA compared with the controls. Of the BDNF receptors, the expression of *NGFR* was significantly increased in T cells from patients with RA (Figure 4C). After adjusting for sex and age, the expression of *CAMK2A* (*p* < 0.001), *MASP2* (*p* < 0.001), *GNG13* (*p* < 0.001), *MUC5AC* (*p* < 0.001), and *NGFR* (*p* = 0.035) was significantly increased in T cells from patient with RA compared with the controls.

### 2.5. Correlation of Clinical Parameters with Expression Level of BDNF-Regulated Genes or BDNF Receptor-NGFR in T Cells from Patients with RA

We analyzed the expression levels of BDNF-regulated genes, including *CAMK2A*, *MASP2*, *GNG13*, and *MUC5AC* or BDNF receptor-*NGFR* in T cells from patients with RA by linear regression analyses. In the simple linear regression analysis, we found that the expression of *GNG3* (*p* = 0.007) and *NGFR* (*p* = 0.012) was positively associated with increasing age in patients with RA. In the multiple linear regression analysis, adjusting for age and sex, RA patients with each 10 year increment of age had a significant 1.45-fold increase (*p* = 0.009; 95% confidence interval (CI) = 1.11–1.91) in *GNG13* expression and 1.40-fold increase (*p* = 0.015; 95% CI = 1.07–1.84) in *NGFR* expression (Table 3).

### 2.6. Functional Studies of BDNF and Its Receptor in Jurkat Cells and Normal PBMCs

**Figure 5:**
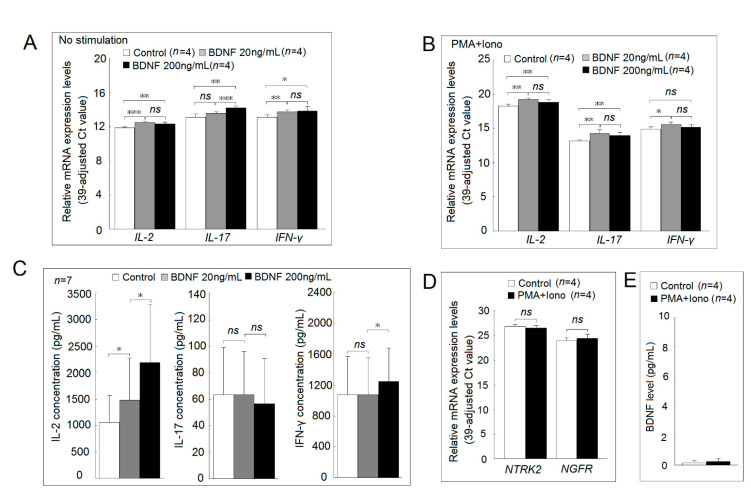
Functional studies of BDNF and its receptors in Jurkat cells and normal peripheral blood mononuclear cells (PBMCs). (**A**) The mRNA expression of *IL-2*, *IL-17* and *IFN-γ* in Jurkat cells with BDNF (0, 20 or 200 ng/mL) for 4 hours. (**B**) The mRNA expression of *IL-2*, *IL-17*, and *IFN-γ* in Jurkat stimulated with phorbol 12-myristate 13-acetate (PMA; 20 ng/mL) + ionomycin (Iono; 1000 ng/mL) plus BDNF (0, 20 or 200 ng/mL) for 4 h. (**C**) Normal PBMCs (1 × 10^6^/mL) were stimulated with 1 μg/mL anti-human CD3 and 1 μg/mL anti-human CD28 plus different concentrations of BDNF (0, 20 or 200 ng/mL) for 24 hours. (**D**) The mRNA expression of *NTRK2* and *NGFR* in Jurkat cells after stimulated with PMA and ionomycin for 4 hours. (**E**) The concentration of BDNF in culture soup of Jurkat cells after cocultured with culture medium or PMA (20 ng/mL) + Iono (1000 ng/mL) for 24 hours (*ns*, not significant; * *p* < 0.05; ** *p* < 0.01; *** *p* < 0.001).

In Jurkat cells, we found that the mRNA expression of *IL-2* and *IFN-γ*, but not *IL-17*, was significantly elevated in those cocultured with a low concentration of BDNF compared with the controls. The mRNA expression of *IL-2, IL-17*, and *IFN-γ* were all significantly elevated in those cocultured with a high concentration of BDNF compared with the controls (Figure 5A). In stimulated Jurkat cells, we found that the mRNA expression of *IL-2*, *IL-17,* and *IFN-γ* was significantly elevated in those cocultured with a low concentration of BDNF compared with the controls. The mRNA expression of *IL-2* and *IL-17*, but not *IFN-γ*, was significantly elevated in those cocultured with high concentrations of BDNF compared with the controls in stimulated Jurkat cells (Figure 5B). In anti-CD3+anti-CD28 activated PBMCs, we found that low concentrations of BDNF increased IL-2, but neither IL-17 nor IFN-γ secretion. High concentrations of BDNF could increase IL-2 and IFN-γ, but not IL-17 secretion (Figure 5C). There were no differences in *NTRK2* and *NGFR* expression between the stimulated Jurkat cells or controls (Figure 5D). We also noted that Jurkat cells did not secrete BDNF either upon activation or not (Figure 5E).

## 3. Discussion

The development of depression in patients with RA is common and is associated with poor treatment response. BDNF is a key mediator of depression. However, few studies have investigated the BDNF levels in patients with RA. Our study showed that serum BDNF levels were higher in patients with RA. Clinically, patients with anxiety, but not depression, and those using biologics were associated with lower BDNF levels. Cheon et al. observed that BDNF was lower in RA patients with depression using serum samples from 154 RA patients with depression and 320 RA patients without depression [6]. However, in our cohort, 96 RA patients suffered from possible depression (HADS-D score ≥ 8), the number of probable depression was only 38. We did not find a significant association between depression and BDNF levels. On the other hand, Suliman et al. found that the serum level of BDNF was reduced in patients with anxiety in a meta-analysis of eight studies with a total of 1179 participants [11]. We did find RA patients with anxiety had lower serum levels of BDNF compared to those without anxiety symptoms. As the symptoms of anxiety and depression are often overlapping, further studies are needed to clarify this issue. The higher serum level of BDNF in patients with RA and the association with biologics used as well as the lack of association between serum BDNF levels and DAS-28 in RA patients, are consistent with a previous study [7]. Steroids or sex hormones could affect the BDNF expression and function [12,13]. We found that 76.6% of the patients with RA were currently using steroids. There was no statistically significant difference in serum BDNF levels between those using steroids or not (17.8 ± 4.3 ng/mL vs. 18.2 ± 3.9 ng/mL, *p* = 0.431). Moreover, the use of steroids was not statistically significantly associated with the serum BDNF levels in the univariate and multiple linear analyses. The impaired hypothalamic-pituitary-adrenal axis in patients with RA might affect the result [14]. Due to the fact that the prevalence of RA is three times more frequent in women than men, up to 78.2% of the RA patients were female in this study. Females also suffered from a higher prevalence of depression. In our study, the percentage of females was comparable in patients with RA and controls (78.2% vs. 67.5%; *p* = 0.114) and the serum BDNF levels remained significantly (*p* = 0.033) elevated in patients with RA compared with the controls after adjusting for age and sex. The result was consistent with a previous report [15]. More studies are needed to clarify this complex issue.

Current evidence showed that there is a close interaction between the immune and nervous systems [8,9]. Several reports have indicated that neurotrophins were involved in the inflammation response of chronic arthritis, including RA [16,17,18]. Neurotrophins, especially BDNF, are known to participate in the pathogenesis of depression [19]. In the immune systems, human T cells, B cells, and monocytes were able to produce BDNF upon activation [20]), and BDNF has been shown to involve in the pathogenesis of experimental autoimmune encephalomyelitis and multiple sclerosis through regulating the survival of autoreactive T cells [21]. With respect to RA, Barthel et al. showed that the two BDNF receptors: *NTRK2* and *NGFR*, were overexpressed in synovial tissue from patients with RA [22]. We found that the mRNA expression of *NGFR*, but not *NTRK2,* was significantly higher in T cells from patients with RA compared with the controls. We further found that patients with RA had elevated serum levels of BDNF, and different concentrations of BDNF had different effects on the phosphorylation of MAPKs. In fact, BDNF binds two receptors; one is the high-affinity receptor NTRK2, also called TrkB, a transmembrane protein that mediates most of its biological functions in neurons. The other one is a low-affinity receptor NGFR, also call p75NTR, for neurite growth and apoptosis [23]. The low concentration of BDNF (20 ng/mL) would affect the Jurkat cells through binding to the high-affinity BDNF receptor, NTRK2, resulting in increased phosphorylation of JNK and ERK and a decreased phosphorylation of p38. The increased phosphorylation of ERK and decreased phosphorylation of p38 in Jurkat cells were consistent with previous reports [24,25]. Increased phosphorylation of JNK could lead to increased phosphorylation of c-Jun. A high concentration of BDNF (200 ng/mL) would affect the functions of Jurkat cells through binding to both NTRK2 and NGFR, resulting in enhanced ERK and p38 phosphorylation in Jurkat cells. However, NGFR binds not only to BDNF but also to NGF, neurotrophin-3 (NT-3), and neurotrophin-4 (NT-4) [26]. NGF is well-known to participate in the immunopathogenesis of RA [16,22,27]. However, the serum levels of BDNF were at least 30-fold higher than NGF, NT-3, or NT-4 [28,29]. We demonstrated that several genes involving inflammation responses, including *CAMK2A, MASP2, GNG13,* and *MUC5AC,* were upregulated in Jurkat cells after cocultured with BDNF as well as T cells from patients with RA. The most important is that BDNF could enhance the mRNA expression of proinflammatory cytokines including *IL-2*, *IL-17,* and *IFN-γ* in resting or stimulated Jurkat cells and increased IL-2 and IFN-γ secretion in activated PBMCs. This finding was consistent with previous studies, which demonstrated that blocking NTRK2 or NGFR could attenuate inflammatory responses [30,31]. We believed this finding could support that increased BDNF levels and differential expression of its receptor in patients with RA could enhance inflammation responses.

The relationship between mood disorder and serum BDNF levels is complex in patients with RA. Patients with depression had lower serum levels of BDNF [32]. Patients with RA also have a high prevalence of depression. However, we were surprised to find that the serum BDNF levels were elevated in patients with RA in our study. Fauchais et al. found that serum levels of BDNF in primary Sjögren’s syndrome patients were significantly higher than those in healthy controls, and the levels were correlated directly with disease activity [33]. Patients with primary Sjögren’s syndrome patients also had a higher risk of depression [34]. We proposed that the local expression and the effect of BDNF in the brain could be critical for the development of depression and anxiety [35]. The effect of increased serum levels of BDNF in patients with systemic autoimmune diseases, such as RA or primary Sjögren’s syndrome, worth further investigation.

## 4. Materials and Methods

### 4.1. Study Subjects

All participants signed informed consent under a study protocol approved by the institutional review board of Dalin Tzu Chi Hospital, Buddhist Tzu Chi Medical Foundation (No. B10603008). The study was performed in accordance with the Declaration of Helsinki. Patients with RA, aged 20 years and above, fulfilled the 2010 American College of Rheumatology (ACR)/European League Against Rheumatism (EULAR) criteria [36] from the outpatient department of rheumatology at the Buddhist Dalin Tzu Chi Hospital were enrolled. The demographic data, medication, and disease activity of patients with RA were recorded. The anxiety and depressive symptoms were recorded with the disease activity score using the hospital anxiety and depression scale (HADS), and we defined depression and anxiety using a cutoff point of 8 in this study [37,38]. In addition, 40 patients with RA and 20 healthy individuals served as a control group for the collection of T cells. Blood samples were collected at least 12 h after the last dose of immunosuppressants to minimize their effects. T cells were purified using anti-human CD3-coated magnetic beads (IMag Cell separation system, BD Bioscience, Franklin Lakes, NJ, USA) according to the method described previously [39].

### 4.2. Flow Cytometry Analysis

The surface expression of two BDNF receptors-neurotrophic receptor tyrosine kinase 2 (NTRK2; also named tropomyosin-related kinase B, TrkB ) and nerve growth factor receptor 2 (NGFR, also named neurotrophin receptor, p75NTR) was determined by stained phycoerythrin-conjugated mouse monoclonal antibody against human NTRK2 (BioLegend, San Diego, California, U.S.), rabbit polyclonal antibodies against NGFR followed by fluorescein isothiocyanate conjugate polyclonal goat-anti-rabbit antibodies (BD Biosciences, Franklin Lakes, NJ, USA) or isotype control (BD Biosciences) analyzing by flow cytometry (FACSMelody, Becton Dickinson, Franklin Lakes, NJ, USA) using FACSChorus software.

### 4.3. Enzyme-Linked Immunosorbent Assay (ELISA)

The concentration of BDNF or proBDNF in the culture supernatants or serum was determined using an ELISA kit (Biosensis, Adelaide, Australia) according to the manufacturer specification. Because the serum levels of proBDNF in some RA patients were undetectable, we used the proBDNF/BDNF ratio instead.

### 4.4. Western Blot Analysis

Western blot analysis was performed as previously described [40]. In brief, the cell lysate was electrophoresed and transferred to a polyvinylidene difluoride (PVDF) sheet (Sigma-Aldrich), then the membranes were nonspecifically blocked in 1% skim milk solution and incubated with the primary antibodies followed by respective HRP-conjugated secondary antibodies. The antibodies used for Western blotting were rabbit monoclonal antibodies against extracellular signal-regulated kinases (ERK)1/2, phospho-ERK1/2 (Thr202/Tyr204), anti-p38, phospho-p38 (Thr180/Tyr182), c-Jun N-terminal kinases (JNK), phospho-JNK (Thr183/Tyr185), anti-c-Jun, anti-phospho-c-Jun (Ser63), and goat-anti-rabbit IgG conjugated with horseradish peroxidase (Cell Signaling Technology, Danvers, MA, USA). Anti-β-actin antibody was used as an internal control (Sigma-Aldrich, St. Louis, MO, USA). Blots were visualized by chemiluminescence reaction (ECL; GE Healthcare, Little Chalfont, UK), and band intensities were measured using Image J (version 1.42; http://rsb.info.nih.gov/ij).

### 4.5. Cell Viability and Proliferation Using the Mitochondrial Dehydrogenase Cleavage Assay

The WST-1 assay was performed according to the method described previously with modifications [41]. After initial treatment, 10 μL WST-1 (Roche Applied Science, Basel, Switzerland) was added to each well, and the plate was incubated for 2 h. The intensity of color formation was detected at OD_450nm_ using an ELISA microplate reader (Anthos Zenyth 3100, Cambridge, UK).

### 4.6. Microarray Analysis

The expression profiles of mRNAs in Jurkat cells cocultured with culture medium or BDNF 200 ng/mL for 48 h were evaluated using microarray analysis. The microarray analysis was performed by Welgene Biotech (Taipei, Taiwan) as in our previous study [38]. In brief, total RNA was extracted by TRIzol reagent (Invitrogen, Carlsbad, CA, USA), then quantified at OD_260nm_ by an ND-1000 spectrophotometer (Thermo Scientific, Wilmington, DE, USA) and quantified using a Bioanalyzer 2100 (Agilent Technology, USA) with an RNA 6000 lab chip kit (Agilent Technologies, Santa Clara, CA, USA). Total RNA was labeled with cyanine 3 (Cy3; Agilent Technologies) dye and hybridized to Agilent SurePrint G3 human V2 GE 8 × 60 K microarray (Agilent Technologies). Microarrays were scanned with an Agilent microarray scanner (Agilent Technologies), and the scanned images were analyzed by Feature extraction 10.5.1.1 software (Agilent Technologies, USA); image analysis and normalization software were used to quantify signal and background intensity for each feature. Raw signal data were normalized by quantile normalization for differential expressed genes discovering.

### 4.7. Measurement of mRNA Expression Levels by Real-Time Reverse Transcription–Polymerase Chain Reaction (RT–PCR)

Jurkat cells or Jurkat cells after activation by phorbol 12-myristate 13-acetate (PMA; 20 ng/mL) + ionomycin (Iono; 1000 ng/mL) were cocultured with BDNF (0, 20 ng/mL or 200 ng/mL) for 4 h. Total RNA was extracted from cells using the Quick-RNA MiniPrep kit (Zymo Research, Irvine, CA, USA) according to the manufacturer’s protocol. RNA concentration was quantified using a spectrophotometer (NanoDrop 1000, Thermo Fisher Scientific, Waltham, MA, USA). Subsequently, mRNA expression levels were quantified by real-time RT–PCR using a one-step RT–PCR kit (TaKaRa, Shiga, Japan) with an ABI Prism 7500 Fast Real-Time PCR system (Applied Biosystems, Waltham, MA, USA) according to the conditions described previously [42]. The relative expression levels of mRNA were defined by the following equation: (39– threshold cycle (Ct) after adjustment based on the expression of 18S ribosomal RNA).

### 4.8. Effect of BDNF on Proinflammatory Cytokines Secretion in Activated Peripheral Blood Mononuclear Cells (PBMCs) from Healthy Individuals

In brief, heparinized venous blood obtained from healthy volunteers was mixed with a 2% dextran solution (mol. wt. 464,000 daltons; Sigma-Aldrich Chemical Company, St. Louis, MO, USA) at a ratio of four parts blood to one part dextran, and the mixture was incubated at room temperature for 30 min. A leukocyte-enriched supernatant was collected and layered over a Ficoll–Hypaque density gradient solution (specific gravity 1.077; Pharmacia Biotech, Uppsala, Sweden). After centrifugation at 250× *g* for 25 min, PBMCs were aspirated from the interface. Then PBMCs (1 × 10^6^/mL) were stimulated with 1 μg/mL anti-human CD3 and 1 μg/mL anti-human CD28 (BioLegend, San Diego, CA, USA) plus different concentrations of BDNF (0, 20, or 200 ng/mL) at 37 °C in 5% CO_2_ for 24 h. After culture, cells were pelleted by centrifugation at 300× *g*, and the supernatant was concomitantly collected and stored at −80 °C for the measurement of cytokines.

### 4.9. Statistical Analysis

Results are represented as the mean ± standard deviation (SD) or *n* (%), as appropriate. Simple and multiple linear regression analyses were performed to obtain correlation coefficients between clinical parameters and serum levels of BDNF, or expression levels of genes in patients with RA. Mann–Whitney U test, Wilcoxon signed-rank test, or Student’s *t*-test was used, as appropriate, to compare different parameters in patients with RA and controls. A *p* value < 0.05 was considered statistically significant. All statistical analyses were conducted using the Stata software (StataCorp, College Station, TX, USA).

## 5. Conclusions

We found that the serum levels of BDNF and T-cell expression of its receptor NGFR were elevated in patients with RA, and decreased serum BDNF levels were correlated with anxiety and biologics used in patients with RA. BDNF promoted inflammatory responses by enhancing the JNK and c-Jun phosphorylation and increased gene expression of *CAMK2A*, *MASP2*, *GNG13*, and *MUC5AC*, in T cells from patients with RA. BDNF could promote the expression of *IL-2*, *IL-17*, and *IFN-γ* in resting or activated Jurkat cells and enhanced IL-2 and IFN-γ secretion in activated normal PBMCs. Targeting BDNF and its signaling pathway may be a novel treatment strategy for RA.

## Figures and Tables

**Table 1:** Univariate and multiple linear analyses of clinical parameters associated with serum levels of BDNF in patients with rheumatoid arthritis (RA) (*N* = 625).

Variable	Univariate Regression Analysis			Multiple Regression Analysis		
	B	(95% CI)	*p*	B	(95% CI)	*p*
Depression (yes/no)	167.23	(−743.59, 1078.05)	0.719			
Anxiety (yes/no)	−1633.44	(−2701.60, −565.28)	0.003	−1660.31	(−2719.44, −601.18)	0.002
DAS-28	−222.15	(−467.34, 23.04)	0.076			
TJC 28	−22.19	(−74.37, 29.98)	0.404			
SJC 28	−91.32	(−169.83, −12.82)	0.023	−52.64	(−131.60, 30.25)	0.220
ESR (mm per h)	−3.66	(−24.65, 17.32)	0.732			
PGA	−8.56	(−21.28, 4.15)	0.187			
CRP (mg/dL)	−158.45	(−426.45, 109.56)	0.247			
Biologics (yes/no)	−920.07	(−1620.78, −219.36)	0.010	−827.25	(−1548.76, −147.37)	0.018
csDMARD (yes/no)	12.08	(−1299.53, 1323.70)	0.986			
Steroid (yes/no)	−312.66	(−1088.67, 463.34)	0.430	−241.91	(−1014.94, 531.11))	0.540
Female	−84.11	(−880.21, 712.00)	0.836			
Age (year)	−30.08	(−54.31, −5.86)	0.015	−12.34	(−47.50, 21.04)	0.449
Educational level						
Below high school	Ref					
High school or above	−33.33	(−713.61, 646.94)	0.923			
Marital status						
Single	Ref					
Married	−728.57	(−1864.78, 407.64)	0.209			
Widowed, divorced or separated	−1144.96	(−2430.36, 140.44)	0.081			
Employment status						
Being employed	Ref					
Unemployed	−601.33	(−1522.66, 320.01)	0.201	−310.42	(−1214.69, 647.55)	0.551
Retired	−1072.76	(−1830.40, −315.12)	0.006	−564.53	(−1554.66, 515.62)	0.325
Income						
High	Ref					
Median	309.26	(−413.22, 1031.73)	0.401			
Low	−147.90	(−1170.72, 874.92)	0.777			
Religious belief (yes/no)	−78.16	(−1568.03, 1411.70)	0.918			
Disease duration ≥5 years (yes/no)	−939.35	(−1767.40, −111.29)	0.026	−626.75	(−1469.76, 218.40)	0.146
Comorbidities	−453.24	(−1153.14, 246.66)	0.204			

CI: confidence interval; CRP: C-reactive protein; DAS28: disease activity score 28; csDMARD: conventional synthetic disease-modifying anti-rheumatic drug; ESR: erythrocyte sedimentation rate; PGA: patient global assessment; RA: rheumatoid arthritis; SJC28: swollen joint count over 28 joints; TJC28: tender joint count over 28 joints. Biologics include etanercept, adalimumab, golimumab, abatacept, tocilizumab, tofacitinib, and rituximab.

**Table 2:** Comparison of demographics and clinical data between patients with rheumatoid arthritis and healthy volunteers.

Variable	Healthy Volunteers (*N* = 20)	Patients with Rheumatoid Arthritis (*N* = 40)	*p*
Age (mean years ± SD)	48.0 ± 6.8	50.6 ± 10.2	0.144
Sex (F:M)	15:5	31:9	>0.999
RF positivity	-	65.7% (26/40)	
ACPA positivity	-	65.7% (23/35)	
DAS28-ESR		3.19 ± 1.05	
CRP (mg/dL)	-	0.56 ± 0.79	
Medication			
Corticosteroids	-	87.5% (35/40)	
Salazopyrine	-	72.5% (29/40)	
MTX	-	80.0% (32/40)	
Leflunomide	-	10.0% (4/40)	
Biologics	-	67.5% (27/40)	

ACPA, anti-citrullinated protein antibody; CRP, C-reactive protein; MTX, methotrexate; RF, rheumatoid factor; SD, standard deviation, - not available; biologics including etanercept, adalimumab, golimumab, tocilizumab, tofacitinib, and rituximab.

**Table 3:** Simple and multiple linear regression analyses for assessing the correlations among different clinical parameters, and expression levels of brain-derived neurotrophic factor (BDNF) regulated genes in T cells of patients with rheumatoid arthritis.

	*CAMK2A*	*MASP2*	*GNG13*	*MUC5A*	*NGFR*
Sex (F/M)	0.144 (0.791)	0.581 (0.414)	−0.380 (0.458)	0.081 (0.841)	0.493 (0.299)
Age (per 10 years)	0.409 (0.064)	0.427 (0.144)	0.549 (0.007) *	0.029 (0.077)	0.504 (0.012) *
Positivity of RF (yes/no)	0.293 (0.538)	0.648 (0.297)	0.616 (0.165)	−0.077 (0.827)	0.013 (0.977)
CRP (per 1 mg/dL)	−0.071 (0.807)	0.037 (0.922)	−0.140 (0.610)	0.082 (0.703)	−0.114 (0.672)
DAS28-ESR	−0.031 (0.887)	0.035 (0.902)	0.109 (0.597)	0.011 (0.946)	0.064 (0.753)
Biologic (yes/no)	−0.209 (0.666)	−0.248 (0.696)	0.236 (0.606)	−0.143 (0.690)	−0.012 (0.979)

DAS28-ESR, Disease Activity Score 28-erythrocyte sedimentation rate; CRP, C-reactive protein; RF, rheumatoid factor. Values are correlation coefficients and (*p* values) from simple linear regression analyses. * After adjusting for age and sex, each 10-year increment of age was significantly associated with a 1.45-fold increase (*p* = 0.009; 95% confidence interval (CI) = 1.11–1.91) in *GNG13* expression and 1.40-fold increase (*p* = 0.015; 95% confidence interval (CI) = 1.07–1.84) in *NGFR* expression among patients with rheumatoid arthritis.

## Data Availability

The datasets analyzed during the current study are available from the corresponding author on reasonable request.

## Abbreviations

BDNF	Brain-derived neurotrophic factor
CAMK2A	Calcium/calmodulin-dependent protein kinase II alpha
CD40	Cluster of differentiation 40
ELISA	Enzyme-linked immunosorbent assay
ERK	Extracellular signal-regulated kinase
GNG13	G protein subunit gamma 13
IFN-γ	Interferon-gamma
IL	Interleukin
Iono	Ionomycin
JNK	c-Jun N-terminal kinase
MASP2	Mannan-binding lectin serine protease 2
MUC5AC	Oligomeric mucus/gel-forming
NGFR	Nerve growth factor receptor
NTRK2	Neurotrophic receptor tyrosine kinase 2
PBMCs	Peripheral blood mononuclear cells
PMA	Phorbol 12-myristate 13-acetate
PVDF	Polyvinylidene difluoride
RA	Rheumatoid arthritis
RT–PCR	Real-time reverse transcription–polymerase chain reaction
STARD13	Star-related lipid transfer domain-containing 13
